# Efficacy of Pyrimethamine/Sulfadoxine versus Chloroquine for the Treatment of Uncomplicated Falciparum Malaria in Children Aged Under 5 Years

**Published:** 2013

**Authors:** W Zheng, H Jiang, Z Xiong, Z Jiang, H Chen

**Affiliations:** 1 Nanchang Center for Disease Control and Prevention, Nanchang, 330038, China; 2 Basic Medical College, Whuhan University, Wuhan, 430072, China; 3 Huadong Research Institute for Medicine and Biotechnics, Nanjing, 210002, China

**Keywords:** Pyrimethamine/Sulfadoxine, Chloroquine, Malaria, Systematic review

## Abstract

The children aged under 5 years from vast African areas badly suffer from falciparum malaria and many of them die of this disease. Therapeutic efficacy of anti-malaria drugs, especially pyrimethamine-sulfadoxine (PS) and chloroquine (CQ) to falciparum malaria is frequently evaluated and reported in recent 10 years. Unfortunately, to date, these widespread materials and researches have not been systematically collected and analyzed. In our study, two investigators were employed to widely and independently gather researches on efficacy of PS vs. CQ mono-therapy of falciparum malaria in children aged below 5 years in unpublished and published databases. Meta-analyses were conducted in categories of PS group and CQ group respectively. Pooled OR of PS vs. CQ was 0.11 (95%CI, 0.05-0.24). PS showed higher therapeutic efficacy to falciparum malaria in less-than-5-year children than CQ. Random model was chosen to analyze for the heterogeneity existence between different studies. Subgroup analyses were performed, but heterogeneity was still presented. Heterogeneity might be caused by different resistance of falciparum malaria to PS and CQ in different settings. Malaria type associated with parasite species, basic information of PS and CQ, and PS & CQ resistant malaria control measures were demonstrated and discussed respectively in detail in this article.

## Introduction

By far, the most important vector borne pathogen infection is malaria, due to one of several *Plasmodium* parasites which is transmitted by the bite of any one of the 50 species of *Anopheles* mosquitoes. Malaria is one of the most important diseases in the world. It exacts an enormous toll social life, medical costs and productivity in tropical regions of Africa, Asia, and Central and South Americas (1–2). In 10 top causes of death all over the world, malaria takes 5th place, following lower respiratory infections, diarrhoeal diseases, HIV/AIDS, and ischemic heart disease (3). Children aged below 5 years old frequently suffer from malaria with high morbidity and mortality. Many deaths in recent year belong to Angola, Benin, Burkina Faso, Cameroon, Central African Republic, Chad, Congo, Côte d'Ivoire, Democratic Republic of the Congo, Guinea, Guinea-Bissau, Mali, Mozambique, Niger, Nigeria, Sierra Leone, Uganda, and Zambia. Mortality in these countries which all come from Africa exceeds 100 persons per 100 000 population per year (4).

### *Plasmodium* Species malaria prevalence

Malaria is caused by the protozoan *Plasmodium*, generally speaking, four species of which infect humans. These species are *P. falciparum*, *P. vivax*, *P. ovale* and *P. malariae*. However, the fifth malaria parasite *P. knowlesi* has raised public health concern (5). Almost all deaths and severe diseases are caused by *P*. *falciparum*. *Falciparum* is the predominant species in most endemic countries, exceptions being part of Southeast Asia and South America where *vivax* is more common (6–11). *Falciparum* malaria can form uncomplicated phase before severe malaria. If falciparum malaria was cured in uncomplicated phase, the mortality and morbidity of severe malaria will be reduced by a large margin.

### Commonly used antimalarial drugs

Many drugs are applied to combat malaria. They are divided into six groups including aryl amino alcohols, 4-aminoquinolines, folate synthesis inhibitors, 8-aminoquinolines, antimicrobials, peroxides. The recent frequently recommended use happens in quinine (QN), mefloquine (MQ) of aryl amino alcohols, amodiaquine (AQ), chloroquine (CQ) of 4-aminoquinolines, pyrimethamine-sulfadoxine (PS) of folate synthesis inhibitors, primaquine (PQ) of 8-aminoquinolines, clindamycin (CL), dox-ycyclinean (D), tetracycline (T) of antimicrobials, and artemether-lumefantrine (AL), artesunate (AS), dihydroartemisinin (DHA) of peroxides. Two or more same or different kinds of them are recommended to use in combination, except CQ therapy of *vivax* malaria only, PS prevention of falciparum malaria in pregnancy and anti-malarial drug mono-therapy of malaria in non-epidemic regions (6–11). Among them, QN, AQ, CQ, PS and artemisinin (AN) are commonly used to treat global malaria. These drugs undergo different developmental history of application. QN had ever been the mainstream for treating severe malaria in children for several years. However, quinine-resistant malaria had been documented in Africa between 1980s and 1990s (12–15). Similarly, CQ had been the mainstay for malaria treatment for the past 40 years. Yet its use had been far and wide limited by the emergence and spread of CQ resistance in most endemic regions, and few countries were unaffected. In 1961, CQ resistance was firstly reported in Colombia (16). The resistance of Sudanese *P. falciparum* to CQ was first reported in Medani (17). In the mid 1970s, low-level resistance to CQ emerged in South Asia, and resistance to CQ increased in this region in the 1980s (18, 19). By 1985, CQ resistance had affected 24 African countries (20). Since then, the proportion of cases of *P. falciparum* malaria which was at least partially resistant to CQ had risen steadily. In major areas, CQ was suspended to be a satisfactory first-line treatment for malaria and alternatives were chosen (21). Current options for the treatment of acute uncomplicated CQ-resistant *P. falciparum* infections in Africa include the use of AQ or PS. This action have led to increasing use of alternative antimalarial drugs, notably PS. Although early studies show full sensitivity to PS, its efficacy for the treatment of symptomatic malaria in children declines after its use for antimalaria, raising concerns about its longevity for the treatment of malaria in children. However, most of malaria-endemic countries in Africa ill afford the more alternatives to CQ or PS (22). AQ is widely available in Africa and also already considered as a possible replacement for CQ in many parts of that continent (23–26). AN is invented in China, it is useful to control malaria. The use of artemisinin, its derivatives and artemisinin-based combination therapies is currently regarded as the best option for the treatment of malaria (27) and has been proposed as a strategy for tackling the problem of drug-resistant falciparum malaria. Nevertheless, cost implications have precluded the quick adoption of artemisinin-based regimes for controlling malaria in most African countries.

Although efficacy failure of PS and CQ to malaria is generally reported, PS & CQ are still extensively used to combat malaria for its low cost, especially in developing countries, such as countries from Africa. In this continent, CQ and PS remain the first-line and second-line therapies of uncomplicated malaria in some areas. In this study, we reviewed effect of PS vs. CQ on uncomplicated falciparum malaria in children aged below 5 years old in recent 10 years through meta-analysis. Before that, we looked back to some basic information about the invention, use history, and resistance of PS and CQ.

### Basic information of PS

The antimalarial efficacy of sulfa drugs was discovered in 1940s. They inhibit the malarial parasites multiplication and development by blocking nucleic acid synthesis (28). In 1950, pyrimethamine was firstly invented by George Hitchings and his comrades at Burroughs Wellcome in the United States. At its primary period of synthesis, it was synthesized as an anticancer agent, and then approved as a drug for the treatment of malaria disease in Britain in 1951 (29). Pyrimethamine and other sulfa drug sulfadoxine differently act on folic acid synthesis. Combination of them could synergistically improve the antimalarial efficacy between them.

Use history and study of PS as an antimaliarial drug were described per decade. During 1970-1979, PS for antimalarial was mainly reported in South East Asia, especially Thailand (30, 31), and then in some African countries, such as Gambia (32). In 1980s, more and more countries used PS to combat malaria disease. These countries included South East countries such as Thailand (33), Burma (34), Cambodia (35) and Malaysia (36), African countries such as Zambia (37) and Tanzania (38), Latin America including Brazil (39) and Colombia (40), North America the US (41), Australia (42, 43) and China (44). Malaria resistance was reported in South East Asia (33, 45), Australia (42, 43), Amazon (39) and east, west and central Africa. Utilization of PS plus other antimalarial drugs for the treatment of malaria parasites had been showed in a few countries (40, 46). From 1990 to 1999, PS was frequently and widely recorded to use in vast Africa, such as Kenya (47), Mozambique (48), Zambia (49), Zimbabwe (50), Tanzania (51), Gabon (52), Gambia (53), Sudan and Nigeria (54). Some Asian countries also used PS to fight against malaria involving Philippine (55), Afghanistan (56), India (57) and Vietnam (58). Besides above malaria endemic area, a few Latin American countries (59) introduced PS to deal with malaria disease. Among this term, PS conferred resistance to malaria parasite in many of African countries (48, 60). Combination of PS and other antimalarial drugs to fight against malaria was demonstrated in major areas (48, 58, 61). Since 2000, PS plus more than one drugs have been developed to evaluate the antimalarial efficacy (22, 62–63). PS resistance also has been reported in many places (64–66). Resistant mechanism (67–69) and the side effects of PS (70–71) have been widely studied and revealed.

### Basic information of CQ

In 1891, dye methylene blue was discovered to be able to kill malaria parasites. During World War I, German scientist used it to synthesize the prototype of antimalarials. In 1932, other dye atabrine was employed to synthesize another antimalarials. German scientists found sontoquine in Tunis, and then sontoquine was modified to be chloroquine. Sontoquine and chloroquine were patented for prophylaxis and control of malaria in the United States in 1941. CQ could quickly control the symptom of susceptible malaria with minimal toxicity. In 1949, CQ was approved as antimalarial by the FDA (29).

CQ experienced different use history, resistance development from 1940 to present. At this period, pharmaceutical distribution of CQ between different populations was substantially researched. In 1940s, some papers reported good efficacy of CQ to control acute attacks of sporozoite-induced *vivax* malaria (72, 73). In the periods of 1950-1959, African countries such as West Africa (74), Nigeria (75), and Congo (76) introduced CQ for controlling malaria. Good effect was displayed everywhere exception being in very few areas (77). During 1960-1969, CQ utilization extended for many areas, from Africa, South East Asia to Europe. However, malaria parasites developed widespread resistance to CQ (78–80). At this time, It was found that combination of CQ and table salt or a few other antimalarials could enhance the antimalarial efficacy of CQ (81). Between 1970 and 1979, CQ for antimalarial efficacy was useless because of high resistance development (82–85). Therefore, other antimalarial drugs were employed to combat CQ-resistant malaria (86–88), or CQ in combination with other drugs to control counterpart (89–90). Portion of resistant mechanism of rodent malaria was exposed. Rodent malaria resistance to CQ correlates with the presence of succinate dehydrogenase activity (91). From the researches among this period, we also know that inadequate CQ dose exposure will cultivate resistance of malaria parasite to CQ (92), CQ has effect on parasite biochemical activity, such as pigment clumping (93) and erythrocytic form (94), special population such as the infant (95–96), the pregnant and the person with other diseases (97) had different manifestation in CQ-resistant malaria, and side effects involving neuromyopathy and retinopathy from CQ were demonstrated (95, 98–99). Among 1980-1989, rather-high-level and multi-*plasmodium* CQ resistant malaria parasites were presented (100–101). Imported CQ-resistant malaria cases were reported in some European countries (102–103). Other ant-imalarials including some new drugs halofantrine, tetracycline and desipramine were used for prevention or treatment of CQ resistant malaria (104–105). From vast drug efficacy evaluation for CQ resistant malaria, it was deduced that the judicious use of existing antimalarials, preferably in combinations, was in an attempt to delay the emergence of resistance; and on aggressive research effort aimed at identifying a new generation of antimalarial drugs. Possible causes of CQ resistance were described as ferriprotoporphyrin IX receptor, calcium channel inhibitors and pH dependence (106–108). Besides above report or research, in this course we also understand that CQ could cause pruritus during the treatment of malaria (109, 110). Between 1990 and 1999, mechanisms of action and resistance of CQ were widely and extendedly demonstrated. CQ resistance has no association with point mutation in the multidrug resistance 1 (pfmdr 1) gene (111) and pfmdr2 protein expression (112). The omega repetitive region of the *P. falciparum* CG2 gene acting as marker for CQ resistance should comfirm under further research (113). After malaria infection, *Plasmodium vivax*-parasitized red blood cells (PRBCs) experiences oxidative stress, and this infection changes the anti-oxidative defense system of the host. When CQ introduced, the anti-oxidative defense system returns near normal levels (114). The remaining researches concerned about CQ resistant malaria treatment and side effects prevention and control (115–116). In 2000s, further research on mechanisms of CQ-resistant malaria was reported. Molecular marker for CQ-resistance was found as pfcrt gene (117). Ferriprotoporphyrin IX (the CQ receptor) dimerization was induced after unmasked lipid promotion. The lipid could be unmasked by aging erythrocyte membrane ghosts from untreated or chloroquine-treated. The process indicated that CQ-induced unmasking of a lipid promoted ferriprotoporphyrin IX dimerization in malaria (118). Different *plasmodium* species had different mechanisms of CQ resistance, that was supported by the example that the molecular events underlying *P. vivax* CQR differed from those in *P. falciparum* (119). From 2010 to present, it was reported in some cases that reemergence of CQ sensitive malaria had happened in some areas such as Kenya and Malawi (120–121). Contrary to the report that CQ resistance has no association with point mutation in the multidrug resistance 1 (pfmdr 1) gene and pfmdr2 protein expression, CQ clinical failures in *P. falciparum* malaria are associated with mutant Pfmdr-1 in Madagascar (122).

### PS versus CQ to combat uncomplicated falciparum malaria in under-5-year children in recent 10 years, meta-analysis

Literature search was performed independently by two investigators (ZW, JH). Literatures that we required were available in grey literature databases(unpublished literature) and published literature databases such as PubMed, British Medicine Association (BMA), Cambridge Science Abstract (CSA), Global health, Conference Proceedings Citation Index (CPCI) and Wanfang data (a Chinese database with published paper and unpublished data that is mainly from conference proceedings).

Studies we screened occurred in Africa except a study performed in Myanmar of the Southeast Asia. These areas endured intense and widespread malaria incidence, especially among children aged less than 5 years. The total amount of 1911 patients, 919 for CQ group, 992 for PS group, with sample sizes ranging from 33 to 683, were included in 10 studies for analysis. 3 studies had less than 100 patients in sample size (123–125). More than 200 children were enrolled to participate in three studies (126–128). Patients of the rest studies recruited were from 100 to 200 (129–132). Axillary temperature of patients limited between 37.5 °C and 39.5°C (or 40°C) in 3 articles (123, 126, 130). The rest of studies had lower limit value of 37.5°C without upper limit value (123, 127–130, 132). Parasite density in blood of patients who were selected varied from 2000/mL to 1000, 000/mL in 5 studies (124, 126, 129–131). Parasite density of ≥1000/mL, 2000/mL were arranged respectively in Smithuis et al. and Basco et al. studies (123, 125). Stivanello et al. and Legros et al. set the same upper limit value of parasite density in enrollment patients at 100,000/mL and the different lower limit value, 1,000/mL for the former and 500/mL for the latter (128, 132). The remaining studies had revealed that parasite density was in the range of 2000–20,000/mL (127). There were 4 articles in which the clinicians or experimenter offered 1.25 mg/kg P +0 mg/kg S body weight for PS group and 25mg/kg CQ for CQ group (128–129, 131–132). In the other studies, 1.25 mg/kg P+25 mg/kg S and 25mg/kg CQ body weight were administrated in PS group and CQ group (123–127, 130). All studies chose the oral method as route of administration (123–132). Days of follow-up were distributed into 4 groups in our screening studies, 28 days in 4 studies (127–129, 131), 14 days in 4 studies (123–124, 126, 132), both 42 days and 21 days in one study (125, 130)( Table 1). Total 241 of 992 patients with PS treatment had total failure in the trials in comparison with 572 of 919 patients with CQ treatment. The pooled odd ratio (OR) of PS vs. CQ mono-therapy of malaria in children aged <5 years in 10 screened studies was 0.11 (95% CI, 0.05-0.24). Compared with using CQ to treat malaria, PS had more effective treatment of this disease. The efficacy of PS treatment was significantly better than CQ treatment (Z=5.55, *P*<0.01). The other statistic indicator of 95% CI of OR whose value range excluded the cut-off value 1 also could be utilized to support this conclusion.

**Table 1:** Characteristics of the studies included in this meta-analysis of PS versus CQ mono-therapy for the treatment of uncomplicated falciparum malaria in children with the age under 5 years

Reference	Setting	No. of population	Age	Axillary temperature	Parasite density (/microliter)	Dose of drug received per child	Follow-up time interval
Guthmann et al.	Angola	79CQ, 79PS	6-59 months	≥37.5°C	2000-100 000	1.25mg/kgP +0mg/kg S 25mg/kg CQ	28 days
Grandesso et al.	Chad	73CQ, 93PS	6-59 months	≥37.5°C	2000-100 000	1.25mg/kgP +0 mg/kg S 25mg/kg CQ	28 days
Basco et al.	Cameroon	33CQ, 64PS	less than 5 years	37.5°C-39.5°C	≥2000	1.25mg/kgP +25 mg/kgS 25mg/kg CQ	14 days
Smithuis et al	Myanmar	15CQ, 19PS	less than 5 years	≥37.5°C	≥1000	1.25mg/kgP +25mg/kg S 25mg/kg CQ	42 days
Menard et al.	Central Africa	22CQ, 57PS	6-59 months	≥37.5°C	2000-100 000	1.25mg/kgP +25mg/kg S 30mg/kg CQ	14 days
Kazadi et al.	Congo	350CQ,308PS	6-59 months	37.5°C-39.5°C	2000-100 000	1.25mg/kgP +25 mg/kgS 30mg/kg CQ	14 days
Radigués et al.	Mali	110CQ, 100PS	6-59 months	≥37.5°C	2000-20 000	1.25mg/kgP +25mg/kg S 30mg/kg CQ	28 days
Abacassamo et al.	Mozambique	85 CQ, 83 PS	6-59 months	37.5°C-40°C	2000–100 000	1.25mg/kgP +25mg/kg S 30mg/kg CQ	21 days
Stivanello et al.	Sudan	114CQ, 103PS	6-59 months	≥37.5°C	1000–100 000	1.25mg/kgP +0 mg/kg S 25mg/kg CQ	28 days
Legros et al.	Uganda	53 CQ, 64 PS	6-59 months	≥37.5°C	500–100 000	1.25mg/kgP +0 mg/kg S 25mg/kg CQ	14 days

**Fig. 1:**
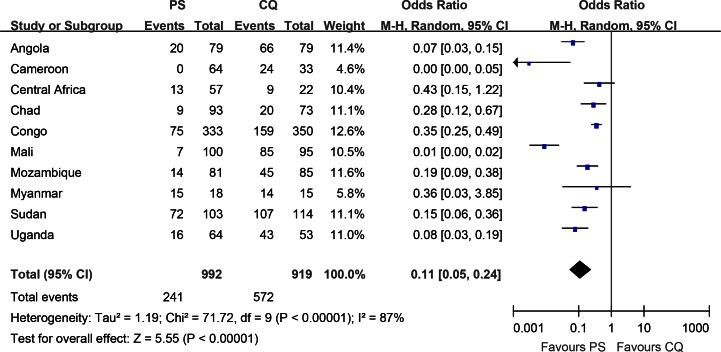
The forest plots demonstrating efficacy of PS versus CQ for the treatment of uncomplicated malaria

Nevertheless, the heterogeneity of these studies occurred (Chi^2^=71.72, *P*<0.1; I^2^=87%) (Fig. 1). The random model was borrowed to calculate and design the combined data from ten studies (Fig. 1). Following the meta-analysis regulation, if the heterogeneity among different studies existed, the subgroup analyses should be used for interpreting the effect difference among subgroups. We conducted the subgroup analyses in term of setting, parasite density, follow-up and drug regime. The subgroups analyses also displayed the heterogeneity between the studies. No reason could be found to explain rationality of the heterogeneity.

Combined data from different studies was useless due to the heterogeneity. We transferred to tell therapeutic efficacy of PS vs. CQ to treat malaria disease one by one. ORs of PS vs. CQ efficacy against malaria in ten studies ranged from 0 to 0.43 (123–132). The lowest OR of PS vs. CQ for the treatment of malaria occurred in Cameroon with value of 0 (95%CI, 0-0.05), highest in Republic of Central Africa with value of 0.43 (95% CI, 0.15-1.22) (123–124). ORs of two studies went beyond 0.33, whose 95%CI included threshold value 1 denoting PS vs. CQ efficacy insignificant difference (124, 125) (view Fig. 1, two lines crossed the central vertical line which represented null hypothesis).The remaining ORs in eight studies changed between 0 and 0.3 (Fig. 1) (123, 126–132)(Fig. 1).

## Discussion and prospective

Many studies reported different effect of PS vs. CQ for fighting against malaria. Some studies showed PS and CQ all had good effect on malaria, some denoted PS moderate efficacy to malaria, and other studies supported mild or little effect of PS and CQ on malaria. Judged from single paper or a study, we could not conclude the real efficacy of PS vs. CQ to combat malaria because of published bias. Until now, only a study for meta-analysis had revealed comparison of PS vs. CQ efficacy for the treatment of malaria in pregnant women and children. This study more concentrated on PS vs. CQ efficacy to women. Children's age did not refer in this study (133). Our meta-analysis and systematic review firstly evaluated the efficacy of PS vs. CQ for the treatment of uncomplicated malaria in children with the age < 5 years. In our study, we screened the objective studies in 340 studies on PS and CQ used for the treatment of malaria disease from PubMed, BMA, CPCI, CSA, Globe Health and Wanfang databases. 10 qualified studies were selected with PS vs. CQ mono-therapy of malaria in less-than-five-year children.

The screened studies were controlled clinical trials, not randomized controlled trials. Drugs were randomly assigned to the enrolled patients from clinicians or experimenter. Nonetheless, we could not find that these study designs included allocation concealment and random sample. There was only one study following blind method using random number. Sample size should be calculated scientifically in a good study design. Most of our screened studies were performed well in sample size calculation regarding of precision, type I error risk, estimated value (failure rate) and loss to follow-up. Blood smear test for parasite density and PCR test for reinfection or recrudescence in late clinical failure/response were aided to assess end/surrogate point of PS vs. CQ effect on children patients. Good laboratory test really reflected the valid clinical response to drugs in patients. Some of our recruited studies involved laboratory quality management. In a word, our enrolled studies designs were not seriously conducted except sample size calculation.

Patients who were characterized by loss to follow-up, reinfection, PCR results inconclusive, or other factors which affected to reflect real efficacy of PS vs. CQ to malaria, were absolutely withdrawn. Total withdrawal didn't exceed 10% of enrolled patients. Patient individuals were recruited to well test efficacy of PS vs. CQ against malaria.

Clinical response of PS vs. CQ to malaria was evaluated by percent of total failure in all patients. Recommended by WHO, anti-malaria drugs response to malaria are subgrouped by total failure which is equal to sum of clinical failure, early treatment failure and late treatment failure (or late clinical failure and late parasitological failure), and adequate clinical and parasitological response (134). Synthetic individuals of total failure and adequate clinical and parasitological response produce total patients. Different response could be assessed by follow-up days, sign and laboratory test. We pooled the data in ten studies for meta-analysis. PS had better efficacy than CQ for treating malaria through meta-analysis. Mean-while, heterogeneity displayed between different studies. Although we stratified different studies and analyzed, heterogeneity also showed. Therefore, OR and other indicators calculated with pooled data was useless. So, we employed systematic review to conduct analysis one by one. Following the rule of sensitivity analysis, we should exclude the studies with small samples and the special studies with large samples. ORs of remaining studies were less than 0.33. All 95%CI in ORs excluded threshold value 1. Hence, we can conclude that PS has more effect than CQ on malaria regardless of its study sites. Nevertheless, the comparative effect of PS vs. CQ in different sites was significantly differently demonstrated. It is implied in our review that different-level drug resistance in different places resulted in PS or CQ having different efficacy to malaria.

Malaria is widely resistant to the first line anti-malaria CQ in African countries, South East Asia and Latin America because of the long-time application for combating malaria (135–141). PS is the second-line drug for controlling malaria. Some countries find its good efficacy to treat malaria (142–144). Some malaria resistance to PS is shown in other countries (145–148). Different areas have different efficacy and resistant level in PS and CQ mono-therapy of malaria. If PS and CQ were used to fight against malaria in an area, history of application and resistant level of two drugs in this area should be systematically supervised and surveyed. Generally speaking, we should consider the first use of PS and CQ to deal with malaria. The other anti-malaria drugs such as AQ, AL, AN, PQ and their derivatives or analogues are secondly utilized for PS and CQ treatment failure in malaria control. Endemic malaria commonly breaks out in undeveloped countries where economy is poor, resource is short of availability, and malnutrition presents here and there. Dearer drugs for vastly curing malaria in these countries are unreasonable. Cheaper drugs-eg, CQ and PS are easily acceptable. Absolute priority must be given to CQ treatment in an area where it isn't used before. Otherwise, we can resort to PS. Therefore, CQ and PS are the first consideration for therapy of malaria although severe resistance occurs in some areas. Nonetheless, application of CQ and PS which have no efficacy to malaria because of resistance should be stopped quickly and widely. The more consumption, the more wastes, and the less curative time in patients. At this moment, other antimalarial drugs must be chosen as replacements for PS or CQ treatment.

As resistance to anti-malarial mono-therapy becomes a serious problem, chemotherapeutic strategy for control of malaria caused by a parasite should be reconsidered. Combination therapy with drugs having different mechanisms of act and biological target in this parasite may be way out (149). It is imminent to make good study design for quickly examining efficacy of two or more anti-malarial drugs in combination. The main aims of the present study are to compare the therapeutic efficacies of mono-therapy using CQ or AQ with those of the combination of CQ or AQ with PS, and to determine which combination is the better efficacy to malaria in the treatment of CQ-resistant infections (22, 124, 150).

Other measures in malaria control are to prevent mosquito bite in addition to intake of anti-malaria drugs. *Anopheles* mosquitoes are main vector of malaria pathogens. At least fifty *Anopheles* species contribute to transmit these pathogens. Population can avoid malaria infection by eradication of mosquitoes. Mosquito prevention and control methods prevail in many articles. At present, many mosquito controls are divided into chemical measurement, biological treatment, habitat management and application of appliance for prevention and control in mosquito bite and annoyance, such as bed-net, mosquito-killer magnet. These control measures are usually used in combination (IPM, Integrated Pest Management). If persons achieved no mosquito bite by these methods, malaria will end among them.

## Conclusion

To date, PS and CQ can also be used to fight against malaria in some areas. However, PS showed higher therapeutic efficacy to falciparum malaria in less-than-5-year children than CQ in our systematic review.
